# XBB 1.5 monovalent booster vaccination stimulates oral mucosal and systemic immune responses in healthy adults

**DOI:** 10.1016/j.vaccine.2026.128346

**Published:** 2026-02-21

**Authors:** Nada Deraz, Michael Payne, Ellen See, Vaishnavi Ragavapuram, Jurgen Bosch, Christopher L. King

**Affiliations:** a Case Western Reserve University, United States; b University Hospitals of Cleveland, Rainbow Babies and Children, United States; c Veterans Affairs Administration, United States

**Keywords:** COVID-19, XBB1.5 monovalent booster, Mucosal immune response, Systemic immune response, Neutralizing antibodies, ACE2 inhibition, Pseudovirus neutralization assay, Spike (S) protein, Secretory IgA (sIgA)

## Abstract

**Background:**

The FDA approved the first monovalent XBB1.5 booster vaccine in September 2023. However, whether this vaccine stimulates mucosal immune responses in the oropharynx, especially neutralizing antibodies, remains understudied.

**Methods:**

We analyzed serum and saliva samples from 28 participants, collected one week before and two to three weeks after the XBB1.5 mRNA vaccination, for neutralizing and binding antibodies to Wuhan and XBB1.5 Spike (S) protein.

**Results:**

We observed a 2.9-fold increase in Wuhan and a 5.0-fold rise in XBB1.5 serum neutralization titers after mRNA vaccination, which correlated with increased binding antibody levels against the S protein variants in serum. We also examined saliva to assess oral mucosal immune responses to vaccination. Vaccination caused a 1.7-fold increase in ACE2 neutralization in saliva against XBB1.5 (*p* < 0.0001) and a 1.4-fold increase against Wuhan (*p* = 0.03). This rise in neutralizing antibody levels in saliva was not associated with the number and timing of previous COVID-19 infections, vaccination status, vaccine type, age, sex, or increases in S-specific IgG in either saliva or serum. Individuals with the largest increase in XBB1.5 neutralization in saliva tended to show a greater rise in S-specific sIgA levels. Depletion of salivary IgA1 or IgG after vaccination revealed that IgA1 was mainly responsible for the increase in ACE2 blocking activity (average reduction in blocking activity with IgA1 depletion = 68%, range 58–81%) compared to IgG (average reduction = 27%, range 0–71%).

**Conclusions:**

The XBB1.5 monovalent vaccine boosts neutralizing antibody levels in blood and mucous membranes, with the largest increase observed against the XBB1.5 variant. This likely reflects the enhancement of cross-reactive antibodies influenced by prior exposure and hybrid immunity, rather than the production of entirely XBB1.5-specific responses. Improved oral mucosal immune responses may reduce the risk of COVID-19 infection and severe illness.

## 1. Introduction

The rapid evolution of the SARS-CoV-2 virus and declining antibody levels after natural infection or vaccination suggest that people may need yearly boosters against new viral variants, similar to recommendations for influenza. Following this approach, the FDA approved an updated XBB.1.5 monovalent booster in September 2023. This is the first monovalent vaccine. Prior vaccines and boosters included only the Wuhan strain, or a bivalent vaccine that included both the Wuhan strain and the Omicron variant, BA.4/BA.5 as an mRNA booster authorized in 2022. The XBB 1.5 booster markedly enhanced neutralization of SARS-CoV-2, including the JN.1 variant, in middle-aged subjects [1] and frail nursing home residents [2]. The XBB 1.5 booster was highly effective against hospitalization by 61% to 76% against the same lineage, but lower against other lineages, such as JN.1, 33% to 46% [3,4].

COVID-19 vaccines provide durable protection against severe illness and death for 4 to 6 months and likely longer [5]. Protection against mild infection and disease provided by vaccines declines more rapidly [6]. This arises because preventing viral transmission likely requires inducing immunity in the respiratory mucosa, which might not be effectively achieved with intramuscular vaccines [7–9]. Studies have shown that COVID-19 mRNA vaccination induces antigen-specific antibodies in nasal washes and saliva in individuals with prior COVID-19, but elicits little or no mucosal response in those without prior infection [10–16]. This vaccine-elicited mucosal immune response is modest and in some cases absent, depending on whether SARS-CoV-2 specific secretory IgA, neutralizing activity, or levels of IgG are measured [11,13,15,17–21]. These differences could be due to variations in the timing between vaccination and sample collection, the interval from the last COVID-19 infection to vaccination, or the methods used for sampling and detection, e.g. saliva or nasal washes. By September 2023, when XBB1.5 vaccination became available, most people had either been exposed to or infected with SARS-CoV-2. Therefore, repeated booster shots may enhance mucosal immune responses. The ability of mRNA vaccines to boost mucosal antibody responses likely depends on the timing and presence of prior SARS-CoV-2 infection, vaccination history, and existing levels of mucosal and systemic immunity.

The leading indicator of protection against SARS-CoV-2 infection in the upper respiratory tract and oral mucosa has been the anti-S secretory IgA (sIgA) levels [22–24]. This immunity, resulting from natural infection, may prevent SARS-CoV-2 from sticking to target epithelial cells, neutralize the spike protein, and stop viral infection and disease [25,26]. The oral cavity and upper respiratory tract each have unique immune systems, called mucosa-associated lymphatic tissue (MALT). Local plasma cells produce multimeric IgA (mostly dimers) from B cells in salivary glands and nasal mucosa (NALT). IgA is absorbed by mucosal epithelial cells via polymeric immunoglobulin receptors on the cell's abluminal side and transported through the cell to be released into the oral and nasal fluids. During transport, IgA acquires a secretory component, becoming sIgA, which protects it from degradation in oral fluids [27,28]. Multimeric sIgA can be up to 15 times more effective than monomeric IgA in neutralizing the virus [29]. Mucosal IgG can also help neutralize the virus in saliva. It is established that acute COVID-19 infection induces neutralizing activity in oral secretions, mainly mediated by sIgA [4,26,37] and confers a period of protection, particularly in the pre-Omicron era [30]. Although IgG can be produced locally in the oral cavity, it is mainly obtained from systemic circulation by transudation and bidirectional neonatal Fc receptor (FcRn)-mediated transport [31,32]. Other antiviral factors in oral secretions, such as lactoferrin, type-I interferons, beta-defensins, and soluble ACE2 [33–36] may also help clear the virus from the upper airways. However, neutralizing antibody responses in the oral mucosa have not been extensively studied in relation to SARS-CoV-2 infection and vaccination. A notable rise in SARS-CoV-2 antigen-specific antibody responses and neutralizing activity in serum has been well documented following mRNA vaccination [37–42]. However, the effect of mRNA vaccines on mucosal immune responses in the upper airways has been less consistent and limited, especially when measuring neutralizing activity [11,13,16,17,42,43]. A consistent finding is that mRNA vaccination produces little [14,20]or no mucosal immune response [11,17,42–44] in individuals who have not been previously infected. Our findings align with several studies indicating that neutralizing antibodies to SARS-CoV-2 are generated in nasal secretions and can be reduced by IgA depletion or increased by IgA enrichment [13,42].

Studies have examined viral neutralization in nasal washes or saliva from the oral mucosa and upper airways [11,13,16,19,42,43]. Early in the pandemic, nasal washes from individuals previously infected with COVID- 19 showed enhanced neutralization activity in a micro- pseudovirus assay, correlating with increased mucosal sIgA but only weakly with IgA depletion in nasal washes [42]. Subsequent studies examined saliva neutralization after the first booster vaccination using semi-quantitative ACE 2- blocking or micro- neutralization assays [11,43]. These studies observed little to no increase in neutralization activity in vaccinated individuals who had not previously contracted COVID- 19. After the booster, individuals with hybrid immunity exhibited enhanced neutralization in mucosal fluids, correlating with S- specific IgG levels both orally and systemically but not with S- specific IgA [19,43]. Another study also found enhanced neutralization in nasal fluids only after vaccination in those with hybrid immunity [13]. The neutralization activity extended to the Delta and Omicron BA 1 variants with mRNA immunization against the Wuhan variant and was primarily mediated by sIgA enriched from nasal sections [13]. However, the mucosal immune response in the oral cavity and upper airways remains understudied following SARS- CoV- 2 infection and vaccination. Recently, several studies reported nasal mucosal immune responses following a monovalent mRNA XBB 1. 5 booster in individuals with hybrid immunity. One study found no significant induction of a mucosal immune response [17], while two others reported increased neutralizing activity after XBB 1. 5 mRNA booster vaccination, with little sIgA induction [16] or no sIgA production [21,45]. The mechanisms mediating this vaccine- enhanced neutralizing activity in the oral cavity in these studies have not been well examined. One possibility is that systemic vaccine- induced IgG is transferred into the oral mucosa, providing the blocking activity [16]. Alternatively, sIgA mediates this blocking activity, but measuring sIgA is insensitive in oral mucosal samples. Whether mRNA vaccination with XBB 1.5 consistently induces neutralizing activity in the oral mucosa, and, if so, what mediates this neutralization, remains to be determined.

We aim to explore the impact of XBB1.5 booster vaccination on healthy individuals of different ages, sexes, and prior CoV-2 exposure to better understand how the booster with a non-Wuhan variant affects mucosal and systemic antibody responses. This will be achieved by measuring vaccine-induced neutralizing antibody function systemically and in oral fluids and determine which mucosal antibodies mediate the enhanced neutralizing activity.

## 2. Methods

### 2.1. Participant recruitment and ethical considerations

Healthcare and laboratory workers participated in the study. After explaining the risks and benefits of the study, written consent was obtained before administering the new XBB 1.5 monovalent COVID-19 mRNA booster. The study was approved by the WCG IRB board (No. 1283160). We collected serum and saliva samples, demographic data, and information about participants' COVID-19 vaccination and infection history from 28 participants within one week before and approximately 2–3 weeks after receiving the booster (see Table 1). Most participants (*N* = 19) also took part in a cohort study examining the impact of acute COVID-19 infection on host immune responses. The records confirmed documented acute COVID-19 infections, identified by PCR, rapid diagnostic test, or a significant increase in anti-N antibodies. The remaining nine participants enrolled in this study reported no history of acute COVID-19 or a confirmed COVID-19 diagnosis by a rapid diagnostic test.

### 2.2. Measurement of Serum antigen-specific IgG and IgA

Blood samples were processed by centrifuging collection tubes at 600 ×*g* for 10 min to obtain serum, which was aliquoted and stored at −80 °C. Participants provided saliva samples after a 30-min fast. Saliva was vortexed for 1 min, then centrifuged at 2500 ×*g* for 10 min at room temperature. A 50× protease inhibitor cocktail (Millipore-Sigma, S8820) was added to the saliva supernatant at 1:50, and samples were stored at −80 °C for long-term preservation. Details of the SARS-CoV-2 binding assay for the Spike (S), Receptor Binding Domain (RBD), and full-length nucleocapsid (N) protein have been described previously [38]. Serum samples were diluted in dilution buffer (1% BSA, 0.05% Tween, DI water, and 1× PBS). Serum IgG dilutions ranged from 3200 to 12,800 for detection of antibodies to the S and RBD proteins, while N-protein dilutions were at 1:400. Diluted samples were added to 96-well plates containing conjugated COVID beads. After incubation and washes, a biotinylated affinity purified goat anti-human Fc for total IgG antibody (Jackson ImmunoResearch) or biotinylated anti-human total IgA mAb (recognized Fc portion, MT20, Mabtech), washed followed streptavidin-PE were added to the assay plates. Plates were run on a MagPix machine (Luminex Corp). Between 100 and 200 beads per antigen were added per well. We required acquisition of more than 40 beads per sample.

The estimated amount of IgG-specific S and RBD units for the Wuhan S protein was determined using a secondary standard from the Frederick National Laboratory, which was calibrated to the WHO standard 20/136 and found to be 1354 BAU/ml [46]. When there were no International Standards for the other antigens involved in binding inhibitory responses, we assigned arbitrary AU values, using our standard as 2823, 5555, and 5555 AUs for BA1, BA5, and XBB1.5, respectively, for the Spike protein.

### 2.3. Saliva Covid19-specific IgG and IgA

Saliva samples were diluted 1:5 for the assay. The Spike-specific assays were performed similarly as described for the serum assays. CoV-specific sIgA was detected using 1 μg/ml Mouse Anti-Human recognizing secretory component of IgA (Millipore Corp clone HP6141), followed by F(ab’)2-Goat anti-Mouse IgG (H + L)-PE (ThermoFisher #12–4010–82). To account for differences in immunoglobulin concentration in saliva, the antigen-specific IgG and IgA levels were normalized by dividing by the total IgG or total sIgA in ng/ml measured in the saliva samples and multiplying by 10^5^.

### 2.4. Total IgG and IgA

For measuring total sIgA, Immulon 4 HBX 96-well microtiter plates (ThermoFisher Scientific) were coated overnight at 4°C with 2 μg/ml Mouse Anti-Human recognizing secretory component of IgA (Millipore Corp, clone HP6141). The plate well content was discarded and washed 3 times with 120 μL of wash buffer (1× PBS, 0.05% Tween) and then blocked with 120 μL of 1% BSA in 1× PBS in 37° for 1 h. After blocking, wells were washed three times with 150 μL of wash buffer, and then 50 μL of samples and standards were added and incubated at 37° for 1 h. Plates were washed as above. Then 50 μL of 0.8 mg/ml (1:1000 dilution) of peroxidase-conjugated AffiniPure F(ab’)^2^ Fragment Goat Anti-Human IgA (Jackson ImmunoResearch, 109–036–011) was added and incubated at 37°C for 1 h. Plates were washed as above. Then 100 μL of BioRad TMB (3,3′,5,5′-Tetramethylbenzidine) followed by 50 μL of 10% sulfuric acid (stop solution). Plates were read at an optical density 450 nm on a Versamax ELISA reader. A similar protocol was used for total IgG. However, plates were coated 2 μg/ml of AffiniPure F(ab’) ^2^ Fragment Goat Anti-Human IgG (Jackson ImmunoResearch, 109–006–097) in 1xPBS, pH 7.4 (Gibco) in a volume of 50 μL. Following the addition of the samples and identical incubations and washes described above, the bound IgG was detected with the 50 μL at a concentration of 0.8 mg/ml (11,000 dilution) of peroxidase-conjugated AffiniPure F(ab’) ^2^ Fragment Donkey Anti-Human IgG, (Jackson ImmunoResearch, 109–036-011). Substrate, stopping, and reading plates were identical to that for IgA.

### 2.5. Neutralizing antibody assays

SARS-CoV-2 pseudovirus neutralization assay used lentiviral particles pseudotyped with spike protein based on the Wuhan and Omicron BA.5 strain as previously described [47]. Serial dilutions of serum range from a lower detection limit of 1:12 to 1:8748. pNT_50_ values are defined as the inverse of the 50% inhibitory concentration value for samples that exhibit pseudovirus neutralization above a previously validated threshold of 80% or higher at the highest serum concentration. Samples that did not meet this threshold were assigned the minimum assay value.

For ACE2 neutralization, serum samples were also assessed using serial dilutions, and pNT_50_ values were defined as the inverse of the 50% inhibitory concentration. Processed saliva samples were diluted 1:10 with dilution buffer (1× PBS, 1% BSA, and 0.05% Tween 20) to a final volume of 50 μL. Saliva (or serum samples at various serial dilutions) is dispensed to wells of a white polystyrene, clear-bottom 96-well assay plate (ThermoFisher, 07–200–566, USA). To capture antibodies to SARS-CoV-2, Magnetic MagPlex microspheres (Luminex Corp) were conjugated with 1 μg of SARS-CoV-2 S protein for the Wuhan, XBB.1, BA5, or BA1 variant, as per the manufacturer's instructions and as described above. 50 μl of conjugated beads per sample were vortexed and sonicated, and then added to the saliva samples and incubated at room temperature for ten minutes with vigorous agitation (500 RPM) and 20 min without shaking. After washing twice, antibody-bound beads were incubated at RT in the dark with 50 μl of 80 ng/ml biotinylated human ACE2 (ACRO Biosystems, AC2-H82E6, Newark, DE, USA) for 10 min with vigorous agitation, followed by 20 min without agitation. Beads were then washed twice as previously described, followed by incubation with 50 μl per sample of R-phycoerythrin-labeled streptavidin (Invitrogen, S21388, USA) diluted 1:2000 in dilution buffer for 5 min with vigorous agitation, followed by 10 min without agitation. Beads were washed twice as described previously, resuspended in 100 μl of dilution buffer, and analyzed with the MagPix multiplex system and xPONENT 4.1 software to acquire data. ACE2 binding values were expressed in median fluorescence intensity (MFI) per sample. To calculate the percentage of ACE2 binding for each sample, the MFI for each sample was divided by the blank (no saliva) MFI, which was arbitrarily set at 100% ACE2 binding. Percent ACE2 inhibition was calculated by subtracting the ACE2% binding values from 100.

### 2.6. IgA1 depletion

Before each use, 1 mL of Jacalin agarose (Thermo Scientific, #20395) packed into a 10 mL chromatography column (Bio-Rad #731–1550) was washed with 3 mL of binding buffer (175 mM Tris-HCl, pH 7.5). After washing, a 1 mL aliquot of the clarified saliva sample (diluted 1:1 with binding buffer) was added to the packed column and allowed to flow through by gravity. The flow-through had the same volume and concentrations of other solutes and proteins as the input sample but contained less IgA bound to the column than the aliquot without IgA1 depletion used as a comparator. The column was then washed with 3 mL of binding buffer. To elute bound IgA1, 3 mL of 0.8 M galactose (in binding buffer) was applied to the column. The eluted IgA was collected and buffer-exchanged into PBS by centrifugation using 10 kDa MWCO protein concentrators (Pierce, #88517). Samples were spun at 4000 ×*g* for 15 min. Three buffer exchanges were performed, and the final volume of each concentrated and exchanged sample was brought up to 200 μL. Post-binding flow-through samples and concentrated eluates were tested for ACE2-blocking activity. IgA1 depletion was repeated in samples with high IgA levels in saliva to ensure complete IgA1 depletion.

### 2.7. IgG depletion

Before each use, 1 ml of Protein G agarose (Roche, #11243233001) packed into a 10 ml chromatography column (BioRad # 731–1550) is washed with 3 ml of PBS. After washing, 1 mL of the clarified saliva sample (diluted 1:1 with PBS) is added to the packed column and allowed to flow through by gravity. The flow-through is collected and stored for future analysis. The column is then washed with 3 ml of PBS. To elute IgG, 1.5 ml of IgG elution buffer, pH 2.6 (ThermoFisher, #21004) is applied to the column. The eluted IgG is collected into 1.5 ml of 50 mM Glycine, pH 8.3, and subjected to buffer exchange with PBS via centrifugation using 10 kDa MWCO protein concentrators (Pierce, #88517). Samples were spun at 4000 ×*g* for 15 min. Three buffer exchanges were performed, with the final volume of each concentrated and exchanged sample resuspended to 200 μl. Post-binding flow-through samples and concentrated eluates are tested in ACE2 assays to assess blocking activity. Columns were washed with 3 ml of binding buffer and stored in 20% *v*/v of ethanol and binding buffer.

## 3. Results

### 3.1. Study population and prior COVID-19 infection status

The median age was 41.5 years, with 60.7% females, 85.7% Caucasians, 7.1% African Americans, and one participant each with South and East Asian backgrounds. Three-quarters of participants had one or more documented COVID-19 infections, with a mean (SD) of 330 ± 207 days from their last infection (DLI) at enrollment. Most participants (71.4%) received a primary vaccination series and two or more boosters, with 53.5% receiving the Pfizer XBB.1.5 booster and 46.5% receiving the Moderna XBB.1.5 monovalent booster. Eight of 28 participants (28.5%) also received the influenza vaccine (Table 1).

We measured anti-N protein levels before and after vaccination to determine whether individuals had a recent asymptomatic infection. According to previous studies, a value greater than 1.5 AU/mL indicates prior COVID-19 infection [48]. Of the seven individuals without a history of COVID-19, all but one (Patient No. 35) had anti-N antibodies below this threshold. Others with a prior COVID-19 infection (*N* = 12) were also anti-N antibody-negative, but these individuals had documented infection more than a year earlier. Notably, some participants seroconverted from negative to positive for anti-N (*N* = 7, bold in Table 2). Three anti-N seropositive individuals before vaccination showed a 3.2 to 8.7-fold increase in anti-N antibody levels. Some individuals reported arm pain or malaise following vaccination, which resolved within two or three days but did not correlate with seroconversion or an increase in anti-N antibody levels. These results suggest that asymptomatic SARS-CoV-2 infections in some participants during the study or more likely vaccination boosted anti-N responses [49].

### 3.2. XBB1.5 monovalent vaccination boosts systemic neutralizing antibody titers and S-specific IgG, but not IgA levels

**Fig. 1.**
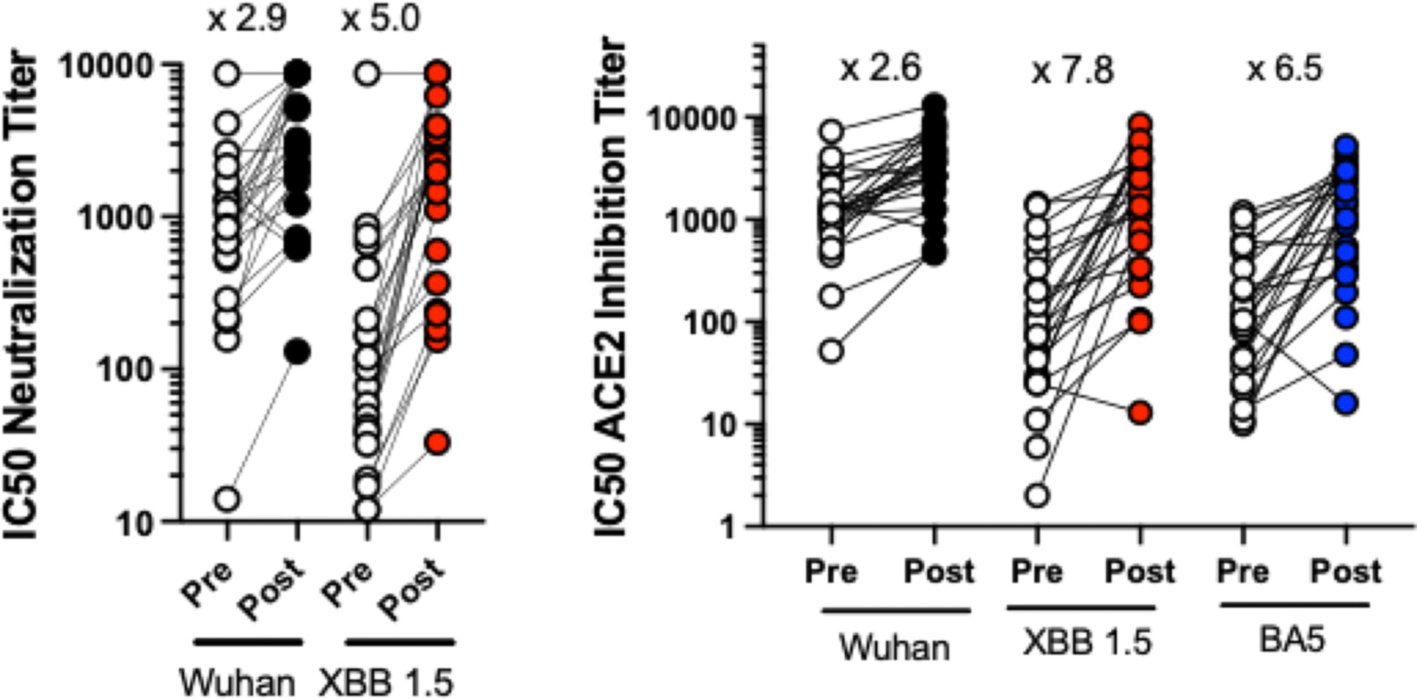
Impact of XBB 1.5 vaccination on viral neutralization titers using pseudovirus assay (left panel) and ACE2 inhibition assay (right panel). Each dot represents a single serum sample tested in parallel with a pre- (white) with a post-vaccination sample (colored dot) for each of the 28 participants. The mean fold increase in neutralization titers pre- and post-vaccination are shown for Wuhan, XBB1.5, and BA5 variants. All increases in neutralization titers are significant to *P* < 0.0001 by the Wilcoxon matched-pairs signed rank test.

**Fig. 2.**
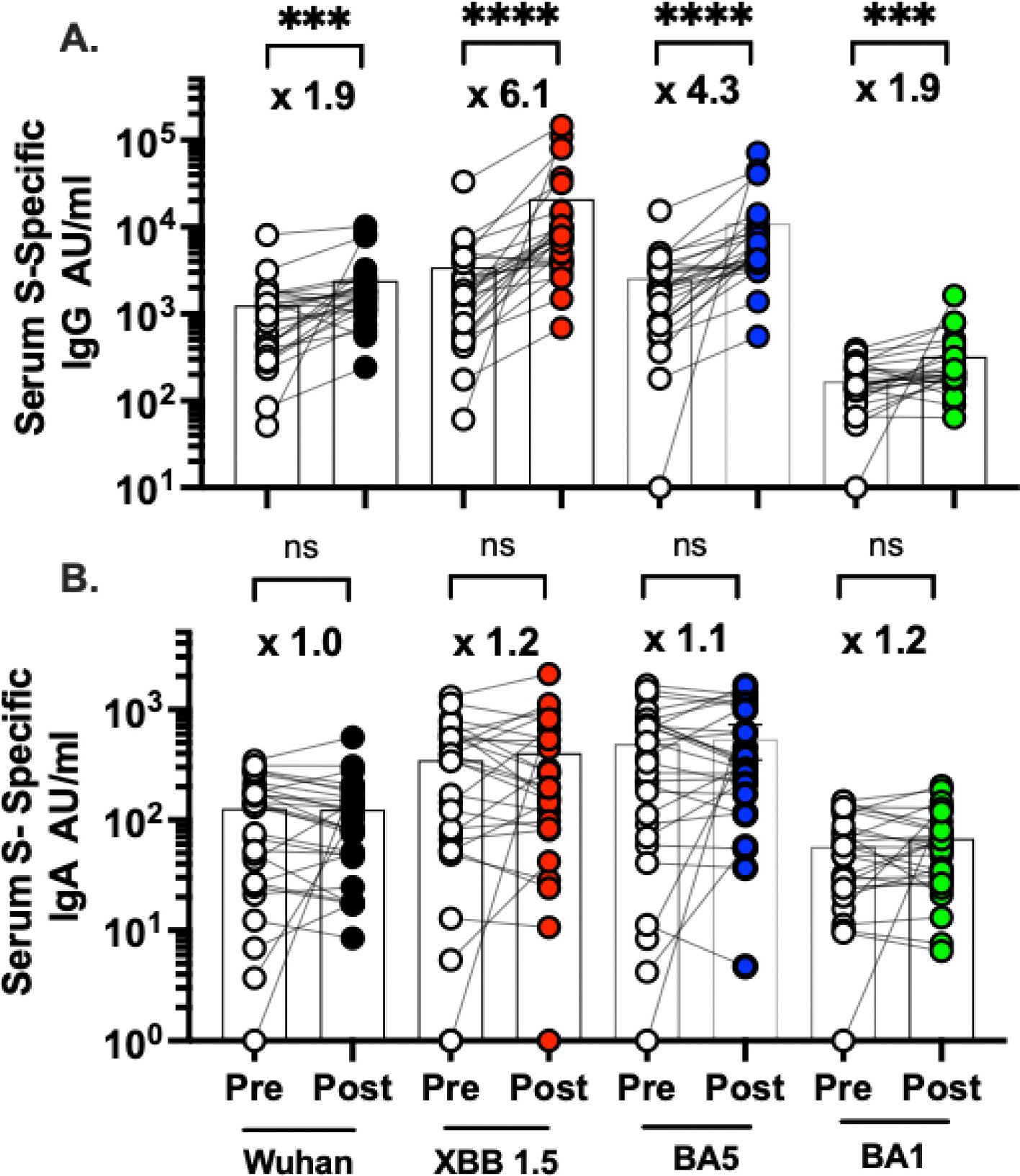
Increased serum IgG (**A**) and IgA (**B**) anti-S protein response using a binding assay following XBB 1.5 booster vaccination. The fold difference in average antibody levels pre- and post- are shown for each variant. Significance was tested between pre- and post-vaccination using the Wilcoxon matched-paired sign rank test. *** *p* < 0.001, **** *p* < 0.0001.

Monovalent booster vaccination significantly enhanced serum-neutralizing antibody titers. Viral neutralization increased by 2.9- and 5-fold for Wuhan and XBB1.5 pre/post titers using the pseudovirus neutralization assay. A similar rise in viral neutralization was observed from 2.6- and 7.8-fold fold to Wuhan and XBB1.5 pre/post titers, respectively, with ACE2 inhibition assay (Fig. 1). The pseudovirus neutralization and ACE2 blocking assays for XBB1.5 correlated well (Spearman *r* = 0.821, *p* < 0.0001) (supplemental Fig. 1). We also measured the impact of vaccination on S protein-binding IgG in serum. Vaccination showed a 1.9-fold (*p* < 0.001) and 6.1-fold (p < 0.0001) increase in Wuhan and XBB1.5 S-specific IgG, respectively (Fig. 2A). The BA1 and BA5 omicron variants showed a similar rise in serum antibody levels. By contrast, vaccination did not increase S-specific IgA overall across the four variants tested (Fig. 2B). Thus, the XBB1.5 booster vaccination elicited a robust increase in S-specific IgG antibody levels against the original Wuhan virus, whereas all the omicron variants tested had no effect on S-specific IgA levels.

### 3.3. XBB1.5 monovalent vaccine boosts saliva ACE2 binding inhibition and S-specific IgG, but not sIgA levels

**Fig. 3.**
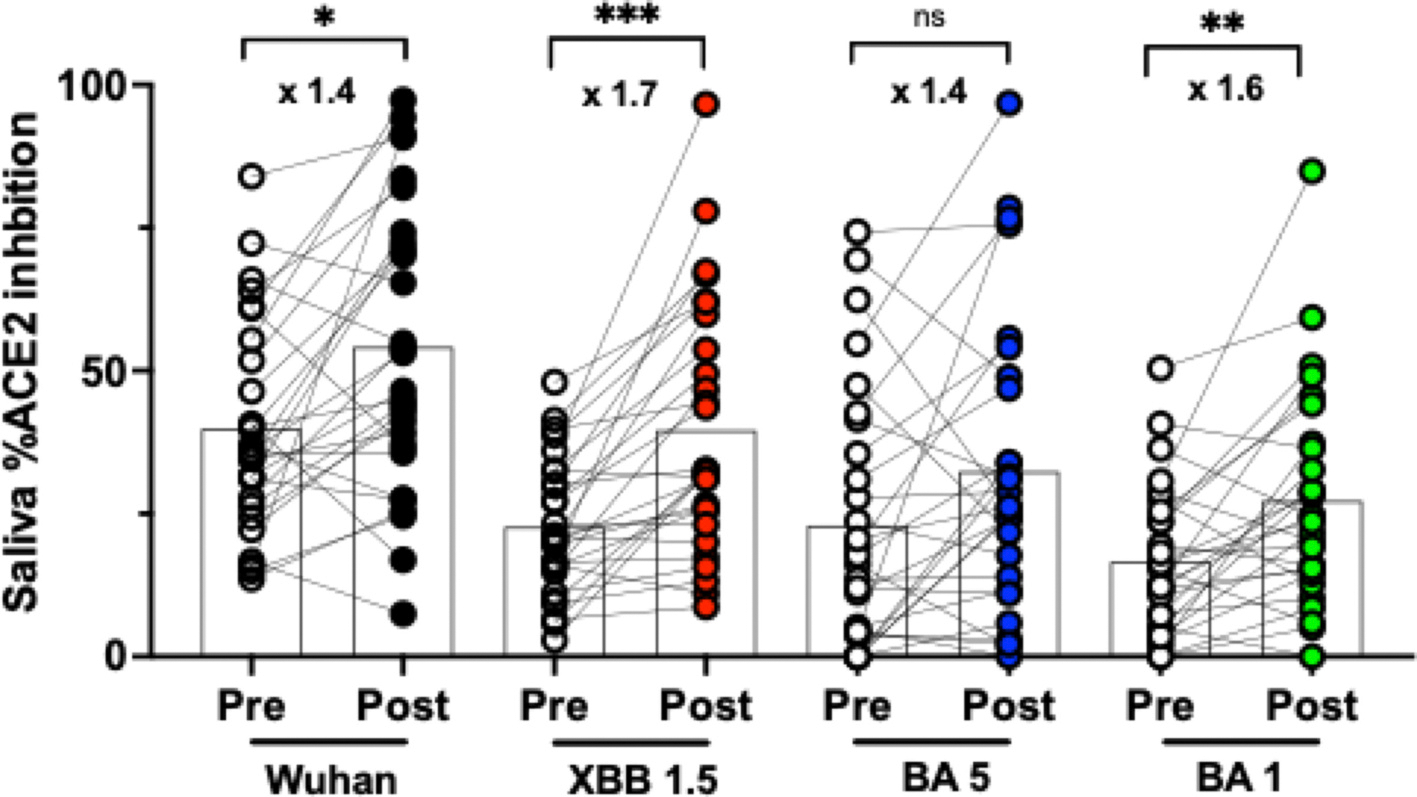
Inhibition of ACE2 binding to different S variants by saliva samples pre- and post-vaccination with XBB1.5 monovalent booster. Paired samples represent the percent ACE2 binding inhibition in participants before and after vaccination. Significance was tested between pre- and post-vaccination using the Wilcoxon matched-paired sign rank test. *p < 0.05, ** *p* < 0.01, *** *p* < 0.001.

**Fig. 4.**
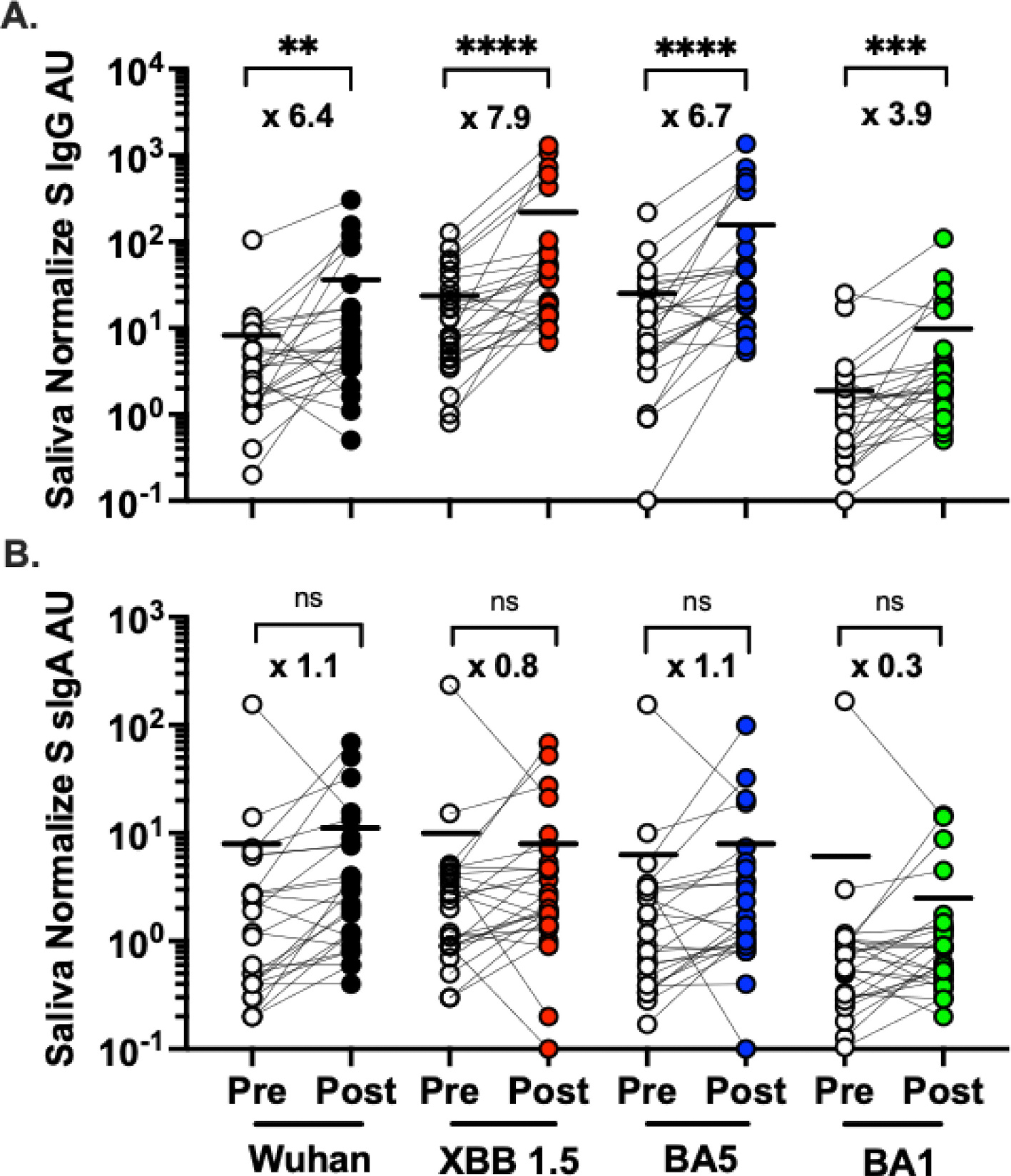
Responses of S-specific IgG **(A)** and IgA **(B)** in saliva after mRNA vaccination. S-specific values are normalized total IgG or IgA measured in saliva. Each circle represents a single serum sample tested in parallel before (open circle) and after vaccination (colored circle) for each participant. Mean fold differences in antibody levels are shown before and after vaccination. Significance between pre- and post-vaccination was tested using the Wilcoxon matched-pairs signed-rank test: **p < 0.01, *** *p* < 0.001, ****p < 0.0001.

Using the ACE2 neutralization assay, XBB1.5 vaccination induced a 1.7-fold increase in binding inhibition in saliva against the XBB1.5 Spike protein (*p* < 0.0001), a 1.4-fold increase against the Wuhan S protein (*p* = 0.03), and a 1.6-fold increase against the BA1 Spike protein (*p* < 0.008, Fig. 3). The 1.4-fold increase with BA5 was not significant. The S-specific IgG level increased significantly in saliva across all variants (Fig. 4A). IgG values were normalized to the total IgG concentration in saliva to account for potential variation in IgG levels. There was a 7.9-fold increase in XBB1.5 S-specific IgG, a 4.2-fold increase in Wuhan IgG, a 6.6-fold increase in BA5 IgG, and a 3.6-fold increase in BA1 IgG. By contrast, there was no overall significant increase in normalized S-specific IgA, although considerable variability in response was observed among individuals (Fig. 4B). Post-vaccination, spike-specific total IgA and sIgA levels in saliva were highly correlated (Spearman's *r* = 0.946, *P* < 0.0001, *N* = 28, supplemental Fig. 2), indicating that most IgA (secondary antibody to IgA heavy chain in the assay) in saliva was sIgA (secondary antibody to secretory protein in the assay). Taken together, these data show mRNA vaccination boosted S-specific IgG but not S-specific IgA in saliva.

We examined the relationship between Spike-specific IgG and IgA levels in serum and.

saliva before and after vaccination (supplemental Table 1). Before vaccination, Spike-specific IgG levels for all variants were significantly correlated between serum and saliva. In contrast, pre-vaccination Spike-specific IgA levels were not correlated, except for antibodies to Wuhan. After vaccination, the relationship between serum and saliva Spike-specific antibody levels changed. Serum Spike-specific IgG was no longer correlated with saliva Spike-specific IgG (Supplemental Table 2). However, a weak correlation emerged between serum and saliva Spike-specific IgA for some variants and not others.

### 3.4. Factors associated with vaccine-induced rise in inhibition of ACE2 binding to XBB 1.5

We examined other parameters that might explain differences in the magnitude of saliva ACE2 inhibition or the increase in Spike-specific IgA to XBB1.5 or Wuhan. We divided the fold increase in saliva neutralization levels against XBB1.5 after vaccination into terciles (Table 2). We assessed whether the number of prior COVID-19 infections, age, sex, vaccination status, vaccine type, and changes in anti-N levels were associated with the rise in saliva S-specific IgA or IgG. None of these parameters was significantly associated with the magnitude of the increase in neutralizing antibody levels. However, individuals in the top tercile of the rise in neutralizing antibody levels also showed the highest increase in S-specific saliva IgA levels (2.6-fold) compared with the other terciles (1.1- and 0.7-fold). Nevertheless, the magnitude of the fold increase in saliva-neutralizing titers and the fold increase in S-specific IgA levels were not significantly correlated (Spearman Rank correlation *r* = 0.31, *P* = 0.14). Therefore, we were unable to identify factors that explain the variability in mRNA-induced neutralizing activity in saliva among the study subjects.

### 3.5. The role of IgA and IgG in mediating neutralizing activity in saliva and serum

**Fig. 5.**
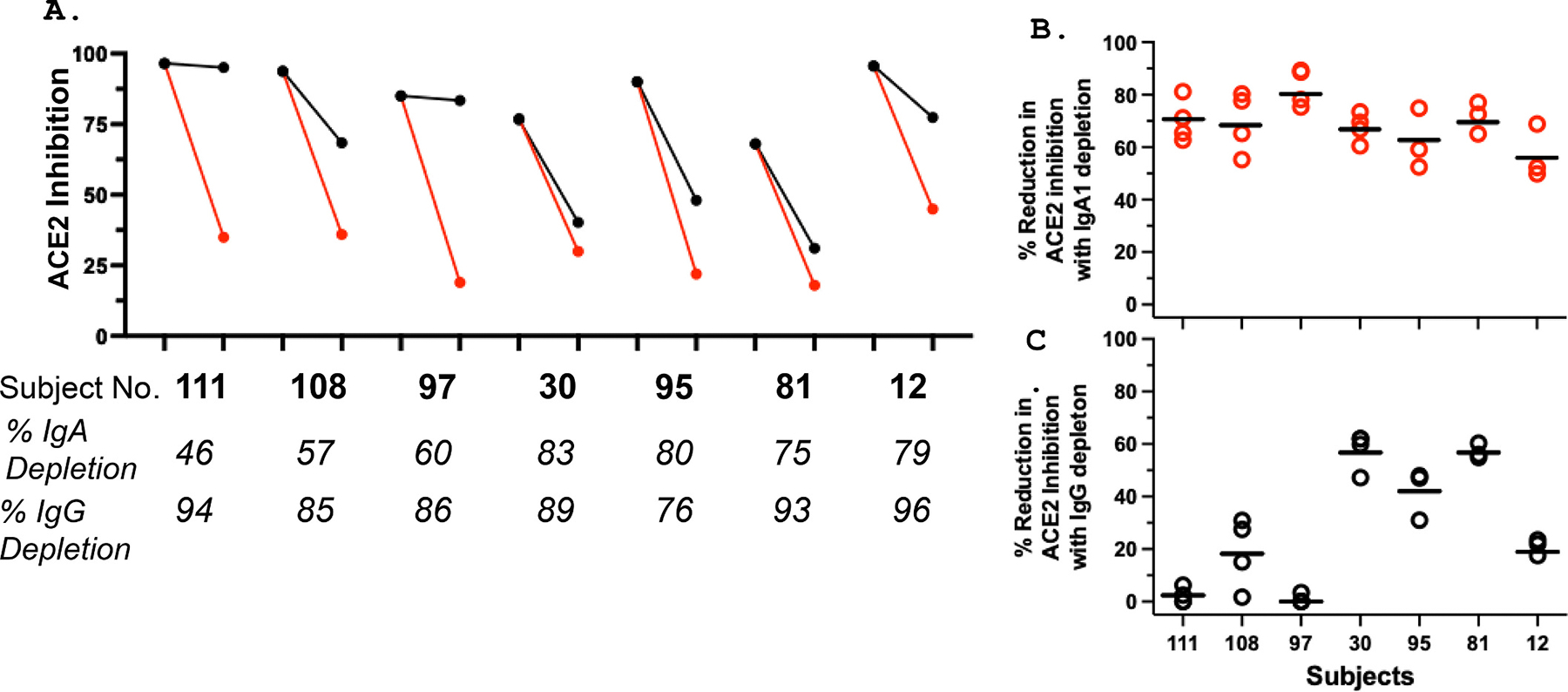
The role of saliva IgA1 and IgG in mediating vaccine-induced XBB 1.5 neutralization in the oral cavity. Seven subjects with more than a 50% increase in ACE2 neutralization after COVID-19 XBB 1.5 booster vaccination were examined to determine whether IgA1 and/or IgG mediated this enhanced neutralization. Panel **(A)** shows the reduction in saliva ACE2 blocking after IgA1 depletion (red lines) and IgG depletion (black lines). Also shown is the percent reduction of S-specific IgA and IgG for each individual. Panels **B** and **C** show the percent depletion of ACE2 inhibition for the results shown in panel A and two to three additional technical replicates. Horizontal bars indicate the means. (For interpretation of the references to colour in this figure legend, the reader is referred to the web version of this article.)

To investigate the roles of IgA and IgG in the vaccine-induced rise in neutralization activity in saliva and serum, we depleted saliva of IgA or IgG after vaccination. Seven participants who experienced the greatest fold increase in neutralization activity (2.7- to 11.1-fold) were examined. On average, 88.5% (range 76–96%) of IgG and 68.6% (range 46–83%) of IgA were removed (Fig. 5A). Jacalin depletes only IgA1, indicating that the remaining IgA is probably IgA2. Depleting IgA1 or IgG from saliva post-vaccination showed that IgA1 was mainly responsible for the rise in neutralization activity (Fig. 5B), with an average reduction in blocking activity of 68% (range 58–81%) with IgA1 depletion, compared with a mean reduction of 27% (range 0–58%) with IgG depletion (Fig. 5C). By contrast, the reduction in neutralization activity to the XBB 1.5 variant following vaccination in serum was primarily mediated by IgG (mean = 85.1%, range = 74.0–100%, Table 3). By contrast, depletion of IgA1 resulted in a mean reduction of 25.2%, range = 8.4–56.7%). Notably, the much higher percentage of IgA depletion in serum with Jacalin compared to saliva is consistent with the fact that most serum IgA is the IgA1 isotype [50]. Taken together, sIgA1 accounts for the majority of the viral-neutralizing activity in saliva but not in serum after mRNA vaccination. By contrast, IgG is primarily responsible for vaccine-induced neutralization activity in serum, but not in saliva.

## 4. Discussion

Our study demonstrated that the XBB1.5 booster significantly increased viral neutralizing activity, both systemically and in the oral mucosa. Similar to other studies [1,17], the XBB1.5 vaccine caused the greatest increase in neutralization activity and antibody binding to the XBB1.5 S protein, with serum increases of 5- to 7.8-fold. We also observed a rise in neutralization and antibody binding to the Wuhan strain, though this was less pronounced because baseline antibody levels against it were higher than against the newer XBB1.5 variant. The pseudovirus and ACE2 neutralization tests in serum showed a strong correlation, supporting the use of the ACE2 binding inhibition test as a proxy for viral neutralization activity in saliva. Using this test, we identified a significant 1.7-fold increase in neutralization against XBB1.5 and a 1.4-fold increase against Wuhan in saliva, along with a smaller but still significant rise against Omicron BA.1 variants. These findings indicate that booster vaccination against a different SARS-CoV-2 variant can enhance a functional antibody response in the oral mucosa of people with hybrid immunity, potentially offering partial protection against infection.

Only half of the participants showed more than a 1.5-fold increase in saliva neutralizing antibodies after vaccination. There was no association with age, sex, race, receipt of Pfizer or Moderna, prior COVID-19 infection, or time since last infection. All participants were healthy, and none were taking immunosuppressive drugs. Although seven individuals did not report COVID-19 infection by late 2023, they likely had exposure to SARS-CoV-2 or had asymptomatic infections. Over one-third of individuals are reported to have asymptomatic SARS-CoV-2 infections [51]. Therefore, hybrid immunity is present in most or all participants three years into the pandemic. We observed seroconversion or antibody boosting to anti-N antibodies in 10 participants during the study. It's unlikely these are asymptomatic SARS-CoV-2 infections in such a short period. More likely, they represent a bystander effect on N-specific B cell stimulation after mRNA vaccination targeting the spike protein. This has been previously documented, especially among individuals with hybrid immunity [49]. Although there was no overall significant increase in salivary IgA after the mRNA booster, individuals with over 1.8-fold increases in neutralizing activity in saliva tended to have higher S-specific IgA levels. Most of this neutralization was due to IgA, shown by the loss of activity after IgA depletion in saliva. Notably, only IgA1 was removed using Jaclin beads. Therefore, the remaining blocking activity in some individuals could be due to the S-specific IgA2 fraction. In a few cases, IgG also contributed to saliva's blocking activity. Some studies suggest that monomeric IgA1 in blood following vaccination may be responsible for neutralization in saliva or nasal secretions [16,43]. However, this seems unlikely here because salivary IgA was mainly secretory, saliva IgA levels didn't consistently correlate with serum IgA, and serum IgA didn't consistently mediate the neutralization observed in the blood after vaccination. By contrast, IgG was primarily responsible for the neutralization activity in serum.

Our results align with some, but not all, studies on whether mRNA can trigger a mucosal immune response. We focus on studies examining mRNA vaccination in individuals with hybrid immunity, a trait common among participants in this study. Some studies measured mucosal immune responses using nasal washes, while others used saliva. Nasal washes tend to be more consistent and display higher antibody levels than saliva [36]. However, saliva is easier to collect, leading to higher participant compliance. Only a few studies measured neutralizing activity in oral fluids after mRNA vaccination. A survey of Swiss subjects supports our findings, showing that mRNA boosts neutralizing activity in nasal secretions, with sIgA playing a key role in this neutralization, especially in individuals with hybrid immunity [6]. They also observed a significant increase in sIgA in nasal fluids only among those with hybrid immunity. We observed a similar trend in vaccinated individuals, with increased sIgA, but only when neutralizing activity rose significantly. They might have seen a greater sIgA response because nasal washes contain higher sIgA levels than saliva. Other studies have shown increased neutralizing activity in saliva [25,28,37] and nasal secretions [27], but did not demonstrate a boost in sIgA or that sIgA mediates this neutralization activity. Instead, they proposed that IgG or IgA isotypes originate from systemic circulation into oral secretions [16,52]. Several factors could explain the variation in mRNA vaccination's ability to induce a mucosal immune response across studies and individuals. Some studies have shown that following mRNA vaccination, the S protein and/or mRNA may persist in circulation for weeks and possibly months in some individuals [53–55]. Therefore, the S antigen could reach mucosal lymphoid tissues, stimulating a mucosal immune response far from the injection site. Other factors may relate to how much SARS-CoV-2 infection stimulates oral mucosal immunity and the expansion of IgA-producing B cells in the mucosa, which then migrate to other mucosal and non-mucosal sites like the spleen or regional lymphoid tissues, where they might be exposed to mRNA from vaccination [56]. This activation enables mucosal B cells to express adhesion molecules, such as α4β7 integrin, CCR9, and CCR10, which help them home to mucosal addressins in the oral mucosa [38]. Whether intramuscular mRNA vaccination can reactivate mucosal memory responses remains to be investigated. If so, encoding chemokine adjuvants in SARS-CoV-2 mRNA vaccines could enhance ligand expression on COVID-specific B cells, increasing their accumulation in the mucosa—a concept considered for HIV vaccines.

Pre-vaccination, serum and saliva Spike-specific IgG levels were correlated, consistent with well-established observations that most salivary IgG originates from the blood via FcRN-mediated transport, primarily through the gingival crevicular epithelium [41]. In contrast, most IgA is produced locally in the nasopharynx-associated lymphoid tissue as sIgA (primarily dimeric) and generally does not correlate with systemic IgA levels (monomers). This is evidenced by the lack of correlation between serum and saliva Spike-specific IgA, for most viral variants. Post-vaccination, serum and saliva Spike-specific IgG levels did not correlate, and there was a weak correlation between serum and saliva Spike-specific IgA. The increase in serum Spike-specific IgG after vaccination may not have had sufficient time to diffuse and reach equilibrium in the oral mucosa. The weak correlation between serum and saliva IgA, especially for Wuhan and XBB1.5, supports the idea that mRNA vaccination stimulated memory B cells in peripheral lymph nodes from prior COVID-19 infections, which then migrated to the oral mucosa.

Protection against COVID-19 infection is strengthened after reinfection [57,58], and repeated infection enhances mucosal immunity [59]. With this increased mucosal immune memory, mucosal memory B cells can populate regional lymph nodes and the spleen [60], and these cells can be stimulated by a systemic SARS-CoV-2 vaccine. Thus, more recent studies might be more likely to show booster vaccine-induced immune responses. Indeed, repeated vaccinations in well-studied Swedish cohorts show a reduction in the detection of mucosal spike IgA, with more vaccinations associated with lower mucosal immune responses [59]. They interpret these results as reflecting improved systemic immunity, which leads to lower viral loads during breakthrough infections and limits the development of mucosal immunity. With the loss of robust mucosal immunity, repeated vaccination may help sustain viral circulation in communities. This further supports the development of mucosal vaccines for SARS-CoV-2 or other respiratory viruses. At present, there are no licensed mucosal vaccines for SARS-CoV-2 in the European Union or the United States. However, five mucosal vaccines have been approved in China, Russia, India, Indonesia, and Morocco [61].

There were limitations to this study. We assessed immune responses at only one time point, which may not capture the full kinetics or durability of mucosal antibody responses. Most vaccine studies examine a single time point after vaccination, which often predicts differences in immune responses when comparing studies that assess oral and systemic antibody responses to COVID-19 at multiple post-vaccination periods [38]. Our study involved a relatively small and diverse cohort, limiting the statistical power to detect associations between demographic or clinical factors and immune outcomes. IgA depletion experiments removed IgA1 but not IgA2, possibly leading to an incomplete assessment of IgA-mediated neutralization. However, most IgA in the oral mucosa is IgA1 [62], although some saliva samples may have contained more IgA2, accounting for incomplete IgA1 depletion in some samples and the failure to eliminate ACE2 blocking in saliva. We measured Spike-specific IgA using antibodies targeting the IgA heavy chain, which would include IgA monomers IgA. The lack of correlation of systemic IgA and with mucosal IgA supports most of IgA is secretory. This approach aligns with prior studies showing that over 90% of IgA in saliva is sIgA [62]. This finding has been confirmed following mRNA vaccination with the SARS-CoV-2 spike protein [14]. Additionally, residual neutralizing activity after IgA depletion could be due to other factors, such as lactoferrin, soluble ACE2, or antimicrobial peptides, which were not tested in this study [63].

In conclusion, the XBB1.5 monovalent vaccine boosts systemic and mucosal viral neutralizing antibodies, primarily against the XBB1.5 variant. Most people now have hybrid immunity to SARS-CoV-2, which has made mRNA vaccines more effective at enhancing mucosal immune responses than earlier in the pandemic. This shows that vaccination can strengthen mucosal defenses by stimulating IgA responses, helping protect against infection. Mucosal immunity in the upper airways could be improved through intranasal vaccines or modifications to mRNA vaccine adjuvants that better target IgA-secreting B cells in the mucosa. Better oral mucosal immunity could lower the risk of COVID-19 progressing to pneumonia and more severe illness, especially in older adults or those with weakened immune systems. Unlike IgG, the exact duration of mucosal protection remains unknown. Therefore, more frequent booster doses may be needed to maintain mucosal immunity and reduce the risk of infection after exposure.

## Supplementary Material

1

## Figures and Tables

**Table 1:** Study population characteristics and anti-N antibody responses.

ID	Age	Sex	Race	Vaccination History	Previous Infections	DLI	Booster	Flu shot	N Protein Pre-Ab; (AU/ml)	N Protein Post-Ab (AU/ml)	Fold difference

97	25	F	C	First booster	1	414	Pfizer	Y	0.55	0.45	0.8
99	29	M	C	First booster	2	992	Pfizer	Y	0.08	1.78*	**23.4**
27	52	M	C	Second booster	1	383	Pfizer	Y	0.82	0.60	0.7
61	71	M	C	Second booster	2	206	Moderna	N	1.65	14.39	8.7
83	27	M	C	First booster	4	256	Pfizer	Y	3.32	3.65	1.1
80	54	F	C	First booster	2	254	Pfizer	Y	0.45	0.81	1.8
100	35	F	C	First booster	1	567	Moderna	N	2.08	2.68	1.3
98	61	F	C	Second booster	1	230	Pfizer	Y	0.41	1.14	2.8
82	76	M	C	Second booster	1	407	Pfizer	Y	0.93	0.96	1.0
101	35	F	C	Second booster	0	NA	Moderna	Y	0.06	0.54	8.5
103	26	F	C	Second booster	3	147	Pfizer	Y	0.02	1.74	**76.1**
12	49	M	AA	First booster	2	542	Moderna	Y	1.21	2.71	**2.2**
86	65	F	C	Second booster	1	209	Moderna	N	1.77	5.69	3.2
30	28	F	C	First booster	0	NA	Pfizer	N	0.10	1.91	**19.8**
35	50	M	C	Second booster	0	NA	Moderna	Y	2.79	0.63	0.2
105	42	F	C	Second booster	0	NA	Pfizer	Y	0.32	0.60	1.9
106	30	F	C	Second booster	1	208	Moderna	N	0.92	1.65	**1.8**
33	48	M	C	Second booster	1	488	Moderna	Y	0.15	0.79	5.3
108	64	F	C	Second booster	1	185	Moderna	Y	0.17	5.49	**32.7**
16	51	F	AA	Second booster	0	NA	Moderna	Y	0.20	4.27	**21.4**
109	36	M	E. Asian	Second booster	1	338	Pfizer	Y	0.54	1.37	2.5
111	26	F	C	First booster	3	127	Pfizer	Y	11.31	5.26	0.5
112	41	F	S. Asian	Second booster	1	492	Pfizer	N	1.07	3.98	**3.7**
92	57	M	C	Second booster	1	158	Moderna	N	2.81	0.91	0.3
115	37	F	C	Second booster	1	212	Pfizer	Y	0.00	0.7	NA
119	73	M	C	Second booster	0	NA	Pfizer	Y	0.02	0.48	22.8
81	25	F	C	Second booster	0	NA	Moderna	N	0.43	0.54	1.2
95	25	F	C	Second booster	1	110	Moderna	Y	3.46	11.03	3.2

Race: C=Caucasian, AA = African American, E. Asian = East Asian, W. Asian = West Asian.

Vaccination History: 1 = no vaccine, 2 = primary series, 3 = primary +1 booster, 4 = primary +2 or more boosters.

DLI: Days from last infection (NA: no documented infection).

Booster type: 1 = Pfizer, 2 = Moderna.

Flu shot: 1 = Yes, 2 = No.

* Greater than 1.5 AU/ml is positive based on > mean + 3SD of pre-COVID samples (*N* = 168).

**Table 2:** Factors associated with vaccine-induced rise in inhibition of ACE2 binding to XBB1.5.

Tercile	Mean±SD (Range) Fold Increase in XBB1.5 neutralization	Median age (IQR)	Sex Female Male (% Female)	Mean±SD (Range) Days since last infection	Mean±SD Days since XBB1.5 booster	Mean±SD No. of prior vaccinations	Mean±SD No. of prior CoV2 infections (range)	Anti-NCP sero-conversion or rise in titer	Mean±SD Fold Rise in S-IgA	Mean±SD Fold Rise in S-IgG	Pfizer/Moderna

**1**	3.9±2.8 (1.7–11.1)	46 (25,53)	7/3 70%	243±118 (110–414)	15.6±2.1	3.7±0.5	1.1±1.0 (0–3)	4 or 10 (40%)	2.6±4.6	3.9±5.0	5/5
**2**	1.5±0.2 (1.2–1.6)	35 (29,45)	4/5 (44%)	444±283 (208–992)	18.3±5.9	3.4±0.5	1.3±1.2 (0–4)	4 of 9 (44%)	1.1±7.5	6.2±3.2	4/5
**3**	0.9±0.2 (0.6–1.1)	48 (37,57)	6/3 (67%)	299±145 (147–492)	19.2±5.2	4.0±0.0	1.0±0.9 (0–3)	3 of 9 (33%)	0.7±5.9	3.5±3.1	5/4

**Table 3:** The Effects of Serum Depletion of IgA or IgG on Systemic Neutralization Activity.

Participant		Wuhan ACE2* blocking (% Reduction in blocking with Ig depletion)		XBB1.5 ACE2 blocking (% Reduction in blocking with Ig depletion)		% IgG Depletion	% IgA Depletion

	undepleted	79.7		77.2			
97	IgA depleted	76.5	(4.1%)	51.0	(33.9%)		95
	IgG depleted	9.4	(88.2%)	8.5	(83.3%)	97	
	undepleted	88.8		79.4			
80	IgA depleted	82.2	(7.4%)	64.0	(19.4%)		93
	IgG depleted	21.4	(76.0%)	16.7	(74.0%)	96	
	undepleted	91.7		80.4			
30	IgA depleted	79.3	(13.5%)	67.3	(16.3%)		81
	IgG depleted	13.0	(85.8%)	9.0	(86.6%)	93	
	undepleted	63.0		50.0			
81	IgA depleted	58.2	(7.6%)	42.0	(16.1%)		87
	IgG depleted	0.0	(100.0%)	0.0	(100.0%)	99	
	undepleted	75.5		66.8			
111	IgA depleted	63.8	(15.6%)	49.9	(25.3%)		95
	IgG depleted	5.7	(92.4%)	5.9	(88.2%)	97	
	undepleted	90.6		77.1			
108	IgA depleted	81.7	(9.9%)	33.4	(56.7%)		82
	IgG depleted	6.3	(93.1%)	4.5	(86.5%)	96	
	undepleted	70.3		83.5			
12	IgA depleted	63.0	(10.4%)	76.5	(8.4%)		97
	IgG depleted	20.3	(71.1%)	17.7	(76.9%)	92	

* Serum samples used at 1:3600 dilution.

## Data Availability

Data will be made available on request.

## Appendix A. Supplementary data

Supplementary data to this article can be found online at https://doi.org/10.1016/j.vaccine.2026.128346.
