# Supplementary material for: XBB 1.5 monovalent booster vaccination stimulates oral mucosal and systemic immune responses in healthy adults
Source: Vaccine. Author manuscript; Available in PMC 2026 Jul 8. (PMC13345680; doi:10.1016/j.vaccine.2026.128346)
Supplement: 1 [file NIHMS2178264-supplement-1.docx]

**Supplementary Table 1:** Correlation of serum and saliva S-specific IgG and IgA before vaccination

| **Serum**  **S-IgG** | *XBB1.5* | **0.74 (***)** |  |  |  |
| --- | --- | --- | --- | --- | --- |
|  | *Wuhan* | **0.66 (***)** | **0.63 (***)** |  |  |
|  | *BA5* | **0.74 (****)** | **0.71 (***)** | **0.76 (***)** |  |
|  | *BA1* | **0.23 (ns)** | **0.58 (**)** | **0.58 (**)** | **0.60 (**)** |
|  |  | *XBB1.5* | *Wuhan* | *BA5* | *BA1* |
|  |  | Normalized **Saliva** S-IgG | | |  |
| **Serum**  **S-IgA** | *XBB1.5* | **0.27 (ns)** |  |  |  |
|  | *Wuhan* | **0.35 (ns)** | **0.53 (**)** |  |  |
|  | *BA5* | **0.33 (ns)** | **0.43 (*)** | **0.23 (ns)** |  |
|  | *BA1* | **0.12 (ns)** | **0.38 (ns)** | **0.07 (ns)** | **0.18 (ns)** |
|  |  | *XBB1.5* | *Wuhan* | *BA5* | *BA1* |
|  |  | Normalized **Saliva** S-IgA  Correlation coefficients represent Spearman’s R. *p<0.05, ** p<0.01, *** p<0.001. | | |  |

| **Serum**  **S-IgG** | *XBB1.5* | **0.23 (ns)** |  |  |  |
| --- | --- | --- | --- | --- | --- |
|  | *Wuhan* | **0.23 (ns)** | **0.20 (ns)** |  |  |
|  | *BA5* | **0.37 (ns)** | **0.31 (ns)** | **0.42 (*)** |  |
|  | *BA1* | **0.39 (ns)** | **0.34 (ns)** | **0.31 (ns)** | **0.33 (ns)** |
|  |  | *XBB1.5* | *Wuhan* | *BA5* | *BA1* |
|  |  | Normalized **Saliva** S-IgG | | |  |
|  |  |  |  |  |  |
| **Serum**  **S-IgA** | *XBB1.5* | **0.34 (ns)** |  |  |  |
|  | *Wuhan* | **0.38 (*)** | **0.58 (**)** |  |  |
|  | *BA5* | **0.33 (ns)** | **0.62 (***)** | **0.60 (***)** |  |
|  | *BA1* | **0.25 (ns)** | **0.36 (ns)** | **0.34 (ns)** | **0.26 (ns)** |
|  |  | *XBB1.5* | *Wuhan* | *BA5* | *BA1* |
|  |  | Normalized **Saliva** S-IgA | | |  |

**Supplementary Table 2:** Correlation of serum and saliva S-specific IgG and IgA after vaccination

**Supplementary Figure 1.** Correlation of serum pseudovirus IC50 neutralization titers with IC50 inhibition titers determined by ACE2 inhibition assay for XBB 1.5 (Spearman r = 0.821, p<0.0001).

**Supplementary Figure 2.** The levels of XBB 1.5 Spike-specific total IgA closely correlate with levels of secretory IgA in saliva from the 28 study subjects following XBB 1.5 mRNA vaccination (r=0.946, P<0.0001, Spearman rank). As described in the methods, total IgA was detected with mAb (Abcam MT30) that recognizes the IgA heavy chain, whereas sIgA was measured with mAb (Millipore Corp, clone HP6141) that recognizes the secretory component of IgA. The levels are reported as relative AU/mL based on the same internal standard.
